# Cannabinoid-Induced Hyperemesis: A Conundrum—From Clinical Recognition to Basic Science Mechanisms

**DOI:** 10.3390/ph3072163

**Published:** 2010-07-07

**Authors:** Nissar A. Darmani

**Affiliations:** Department of Basic Medical Sciences, College of Osteopathic Medicine of the Pacific, Western University of Health Sciences, Pomona, CA, USA; E-Mail: ndarmani@westernu.edu; Tel.: +1-909-469-5654; Fax: +1-909 469 5698

**Keywords:** cannabis, hyperemesis, CB _1_ receptor, pharmacokinetic, pharmacodynamic

## Abstract

Cannabinoids are used clinically on a subacute basis as prophylactic agonist antiemetics for the prevention of nausea and vomiting caused by chemotherapeutics. Cannabinoids prevent vomiting by inhibition of release of emetic neurotransmitters via stimulation of presynaptic cannabinoid CB_1_ receptors. Cannabis-induced hyperemesis is a recently recognized syndrome associated with chronic cannabis use. It is characterized by repeated cyclical vomiting and learned compulsive hot water bathing behavior. Although considered rare, recent international publications of numerous case reports suggest the contrary. The syndrome appears to be a paradox and the pathophysiological mechanism(s) underlying the induced vomiting remains unknown. Although some traditional hypotheses have already been proposed, the present review critically explores the basic science of these explanations in the clinical setting and provides more current mechanisms for the induced hyperemesis. These encompass: (1) pharmacokinetic factors such as long half-life, chronic exposure, lipid solubility, individual variation in metabolism/excretion leading to accumulation of emetogenic cannabinoid metabolites, and/or cannabinoid withdrawal; and (2) pharmacodynamic factors including switching of the efficacy of Δ^9^-THC from partial agonist to antagonist, differential interaction of Δ^9^-THC with Gs and Gi signal transduction proteins, CB_1_ receptor desensitization or downregulation, alterations in tissue concentrations of endocannabinoid agonists/inverse agonists, Δ^9^-THC-induced mobilization of emetogenic metabolites of the arachidonic acid cascade, brainstem *versus* enteric actions of Δ^9^-THC, and/or hypothermic *versus* hyperthermic actions of Δ^9^-THC. In addition, human and animal findings suggest that chronic exposure to cannabis may not be a prerequisite for the induction of vomiting but is required for the intensity of emesis.

## 1. Introduction

The association of chronic cannabis use with cyclic-type vomiting syndrome (CVS) in adults is an obscure phenomenon which has recently been recognized by clinicians following publication of a number of case reports as well as three clinical series [1,2,3,4,5,6,7,8,9,10,11,12,13,14]. CVS is a disorder characterized by recurrent, self-limited episodes of severe nausea and vomiting interspersed with symptom free periods [15]. While CVS has been mainly studied in pediatric patients, this enigmatic syndrome represents a continuum affecting all ages, including young and middle aged adults. Affected patients exhibit a stereotypical pattern of multiple episodes of vomiting with frequent visits to emergency departments for relief of nausea, vomiting and dehydration. The clinical feature of CVS can be divided into four phases: (i) the interepisodic “well phase” that persists from weeks to months between vomiting episodes when the patient is relatively symptom free; (ii) the “prodromal nauseous phase” of varying intensity that encompasses the time when the patient begins to sense the approach of vomiting and can last from minutes to hours; (iii) the “emetic phase” is characterized by intense, persistent nausea with repeated episodes of vomiting which may persist from hours to 10 days, and (iv) the “recovery phase”, begins with termination of vomiting, and ends with hunger and tolerance of oral intake. The accompanying signs include anorexia, retching, increased salivation, abdominal pain, headache, pallor, listlessness, photophobia and phonophobia. Triggers of acute episodes of CVS include infections, psychological stress, motion sickness, lack of sleep, physical exhaustion and certain foods. Co-existing conditions in adults with CVS include migraine headaches, psychiatric disease, gastroesophgeal reflux disease, irritable bowel syndrome, gallbladder disease, insulin-dependent diabetes mellitus and chronic marijuana use [15].

The seemingly rare cannabinoid hyperemesis was originally reported from Australia in 2004 [1] but appears to be potentially much more common, as evidenced by the cited recent reports, each discussing one or several cases from the Netherlands, New Zealand, Spain, England and the USA. The phenomenon is now referred to as cannabis hyperemesis syndrome, which manifests with recurrent intense nausea, intractable vomiting and abdominal pain. The detailed phasic nature of cannabinoid hyperemesis syndrome is similar to that described for CVS, but often it is accompanied with compulsive hot bathing (or hot showers) which seems to temporary relieve patients’ symptoms. Relief of gastrointestinal symptoms appears to be temperature-dependent since the hotter the water, the better the antiemetic effect [1,14]. Taking hot baths or showers is a learned behavior and may not be present at the initial presentation. However, once the behavior develops, baths/showers may last for hours and may be repeated up to 20 times per day [14]. The vomiting episodes are cyclical, occurring every few weeks or months and can be preceded by a period of intense morning nausea. The vomiting becomes bilious and culminates in intractable retching which may last for hours. Most episodes resolve within 48 hours, but some may last several days [14]. However, unlike the other forms of CVS, patients suffering from cannabinoid hyperemesis are not likely to have a history of migraine headache but suffer from the peculiar desire for hot showers or baths. The empirical strong association between the syndrome and chronic cannabis use is evidenced by the cessation of the syndrome following cannabis discontinuation in most patients, and the recurrence of the syndrome with cannabis challenge. In fact other than stopping cannabis use, as yet there is no proven treatment [14].

The hyperemetic activity appears to be an enigma since both phyto- (e.g., Δ^9^-THC or Δ^8^-THC) and synthetic (nabilone, levonantradol, or nonabine)-cannabinoids possess significant antiemetic activity both in the clinic and in animal models of emesis [16,17]. Unlike most antiemetics which behave as antagonists of their corresponding emetic receptors, cannabinoids act as agonist antiemetics. The mechanisms by which Δ^9^-THC and its structural analogs produce their antiemetic effect was initially revealed in the least shrew [18] after the identification and cloning of at least two G-protein-coupled receptors called cannabinoid CB_1_ and CB_2_ [19,20]. While the CB_1_ receptor is expressed in the neurons in the CNS, the CB_2_ receptor is often localized in lymphoid tissues in the periphery. Animal models of emesis have revealed that cannabinoids behave as broad-spectrum antiemetics and prevent emesis by stimulating cannabinoid CB_1_ receptors [17]. Endogenous ligands for cannabinoid receptors have also been discovered. To date at least two well-investigated endocannabinoids are recognized, *N*-arachidonoylethanolamide (also called anandamide) and 2-arachidonoylglycerol (2-AG), in both the brain and the gut. Several pathways exist for their formation and catabolism. Following their cellular reuptake, anandamide is metabolized via fatty acid amide hydrolase (FAAH), and 2-AG via monoacylglycerol lipase (MAGL). 2-AG is also metabolized to some extent by other hydrolases in addition to FAAH [19]. Anandamide has the highest affinity (the ability of a drug to bind its receptor *i.e.*, the chemical forces that cause the drug molecule to associate reversibly with its receptor), whereas 2-AG has the greatest efficacy (the strength of a drug that produces a maximal effect by forcing a proportion of available receptors into their active conformational state) for cannabinoid CB_1_ and CB_2_ receptors. Retrograde signaling is an important aspect of cannabinoid function wherein, upon postsynaptic stimulation, endocannabinoids are synthesized on demand in postsynaptic neurons, and diffuse back to presynaptic nerve terminals to stimulate CB_1_ receptors and thus inhibit neurotransmitter release [21]. Unlike classical neurotransmitters such as dopamine or serotonin, endocannabinoids are not prepackaged and stored into vesicles.

Since Δ^9^-THC and related cannabinoid CB_1/2_ receptor agonists have broad-spectrum antiemetic efficacy via stimulation of cannabinoid CB_1_ receptors, the paradoxical mechanism(s) by which cannabis hyperemesis occur are currently not understood. At the inception of clinical recognition of the phenomenon, the basic science knowledge of cannabinoids was utilized to provide a number of conventional explanations for the hyperemesis [1]. However, the possible pharmacological mechanism(s) of the emetic activity of cannabis appear to be much more complex and befit Sir Winston Churchill’s famous political remark “it is a riddle wrapped in a mystery inside an enigma”. Thus, in the current review the proposed traditional hypotheses are further expanded, while others are excluded. Where possible, more contemporary pharmacokinetic and molecular pharmacodynamic explanations with examples are offered:

## 2. Pharmacokinetic Factors

Pharmacokinetics is the study of what the body does to a drug following its administration. Pharmacokinetics is concerned with the processes of drug absorption, bioavailability, distribution, metabolism and excretion which ultimately determine medicine’s duration of action. A number of investigators support Allen and co-workers’ proposal [1] that pharmacokinetic factors contribute to cannabinoid-induced hyperemesis syndrome since several components of cannabis possess long half-lives and are highly lipid soluble, and thus can accumulate in the brain upon chronic exposure [1,8,10,13]. Indeed, the marijuana plant contains over 400 chemicals of which 60 have cannabinoid structure [22]. Thus, it is possible that instead of Δ^9^-THC, other lipid-soluble components of cannabis such as the non-intoxicating cannabidiol, could induce vomiting, since high doses of this compound have been reported to cause emesis in the house musk shrew [23]. Alternatively, certain subjects may have a genetic variation in their metabolic enzymes (e.g., cytochrome P450 enzymes such as CYP2C9, CYP2C19 and CYP3A4) that may result in excessive levels of a metabolite(s) of the diverse cannabinoids found in the cannabis plant which could promote vomiting [13,24]. In fact, the complexity of the subject can be highlighted by just considering the metabolic products of one component of cannabis, Δ^9^-THC. Although it is well known that Δ^9^-THC is mainly biotransformed to 2 metabolites (11-OH-THC and THC-COOH) in the human body, in reality more than 100 minor metabolites of Δ^9^-THC can be identified [24]. Thus, accumulation of one or more of such cannabinoids and/or their metabolites following chronic cannabis exposure in some genetically- and/or renally-compromised individuals could cause vomiting. 

## 3. Pharmacodynamic Factors

Pharmacodynamics is the study of the physiological effects of drugs on the body and is concerned with: (1) the mechanisms of drug action at the receptor and signal transduction levels, and (2) the relationship between drug concentration and the effect produced following drug-receptor interaction(s). Changes in both drug pharmacodynamic factors and receptor parameters can influence the final response produced in an organism. 

### 3.1. Cannabis Withdrawal

In terms of pharmacokinetic/pharmacodynamic changes, Allen and coworkers [1] had proposed that abrupt cessation from prolonged cannabis use may induce hyperemesis in affected patients. This hypothesis is further supported by clinical findings, in that patients experiencing cannabis withdrawal syndrome can experience nausea, vomiting, diarrhea and stomach ache among other responses such as anxiety and depression [25]. Additional support comes from animal studies since rapid precipitation of withdrawal in Δ^9^-THC-tolerant dogs by moderate doses of the cannabinoid CB_1_ receptor antagonist SR141716A (acomplia; rimonabant) can cause profound vomiting [26]. However, at first glance, withdrawal-induced hyperemesis can be discounted since Δ^9^-THC has a long half life in humans and furthermore the affected patients continued their cannabis use up to the onset of hyperemesis [1]. Moreover, these patients were not experiencing other major symptoms of a sudden cannabinoid withdrawal prior vomiting. In addition, resumption of cannabis use following recovery from hyperemesis still led to profound vomiting in these patients. 

### 3.2. Cannabinoid Efficacy and Intrinsic Activity

The ability or strength of a drug to produce a maximal effect following binding to its appropriate receptor is called efficacy [27]. Thus, efficacy is the proportion of available receptors that are forced into their active conformation by an agonist to produce a maximal effect. Efficacy has limits ranging from 0 for competitive silent antagonists to 1 for full agonists, and to −1 for full inverse agonists. Partial agonists’ efficacy values lie between 0 to 1, while efficacy values for partial inverse agonists may range between −1 to 0. Efficacy of a drug is dependent upon its intrinsic activity and the total number of receptors available. Intrinsic activity is a property of the drug molecule itself and is the amount of stimulus a drug molecule applies to a single receptor, and does not vary among different tissues [27]. In contrast, efficacy varies among tissues because it is dependent upon receptor density, which in turn varies among different tissues. Contemporary pharmacodynamic findings in terms of agonist efficacy indicate that Δ^9^-THC acts as a partial agonist on cannabinoid CB_1_ receptors and in some test systems it can antagonize the effects of full agonists on these receptors [28]. Thus, following chronic use and at high tissue concentrations, the antagonist nature of Δ^9^-THC may surface, which could precipitate a sudden withdrawal in some “sensitive” patients in a manner similar to that already described for rimonabant in Δ^9^-THC-dependent dogs [26]. Furthermore, the firing rate of neurons may also determine the partial agonist/antagonist nature of Δ^9^-THC since this euphoriant displays a state-dependent switching from agonist to antagonist, which could account for its complex actions *in vivo* [29]. Because presynaptic cannabinoid CB_1_ receptors behave as heteroreceptors on the terminal ends of a variety of excitatory and inhibitory neurons and control the release of their corresponding neurotransmitters [30], the nature of intrinsic activity (*i.e.* the amount of stimulus a drug applies to a receptor) of Δ^9^-THC becomes paramount. Indeed, when Δ^9^-THC acts normally as a partial agonist (efficacy < 1), it can inhibit the release of neurotransmitters via activation of presynaptic cannabinoid CB_1_ receptors [31], but probably not as much as full CB_1_ receptor agonists (efficacy = 1). On the other hand, at large doses, its possible antagonist action (efficacy = 0) will block CB_1_ receptors and thus would potentially promote the release and turnover of one or more emetogenic transmitters such as serotonin, dopamine or substance P. This would induce emesis as it has been demonstrated in the case of the cannabinoid CB_1_ receptor antagonist rimonabant [32,33]. Furthermore, at the G-protein level, action of lower doses of cannabinoids is thought to involve the stimulatory Gs protein, while higher doses of cannabinoids activate the inhibitory Gi protein [34]. The paradoxical biphasic emetic/antiemetic effect of Δ^9^-THC is not unique to vomiting since cannabinoids produce similar inhibitory/stimulatory actions on spontaneous locomotor activity, rearing and circling behaviors [35,36], cortical evoked responses [37], and anxiolytic/anxiogenic effects [38]. 

### 3.3. CB_1_ Receptor Desensitization and/or Down-Regulation

Other unexplored but essential pharmacodynamic factors that could influence hyperemesis following chronic exposure either to Δ^9^-THC or to cannabinoid CB_1/2_ full agonists, are the degree of presynaptic CB_1_ receptor desensitization and/or reduction in receptor density, either of which could influence the on-demand neuroprotection mediated by endocannabinoids [39]. In fact, animals treated chronically with cannabinoid agonists rapidly develop tolerance to the effects of cannabinoids since cannabinoid receptor expression levels down-regulate [40] and cannabinoid-activated signal transduction mechanisms become profoundly desensitized [41]. Since an agonist’s efficacy is dependent both on its intrinsic activity as well as on the number of functional receptors present in a given tissue, the partial agonist nature of Δ^9^-THC can transform to antagonist action in the presence of reduced functional CB_1_ receptors. In addition, the tissue concentrations of endocannabinoids undergo significant changes following physiological and pathological stimuli [42,43]. Thus, induced alterations in cannabinoid agonist efficacy, or in the density of functional CB_1_ receptors present, or in the tissue concentrations of endocannabinoids, have the potential to affect the endocannabinoid system. Indeed, endocannabinoids as retrograde messengers are synthesized in response to increases in postsynaptic intracellular Ca^2+^ concentration and travel backwards across the synapse where they transiently inhibit the release of either the inhibitory GABA or the excitatory glutamate neurotransmitters. Consequently, endocannabinoids suppress excitotoxicity via presynaptic cannabinoid CB_1_ receptors. Since chronic cannabis exposure causes desensitization and/or down regulation of CB_1_ receptors, it may therefore induce a threat to the effectiveness of the endocannabinoid feedback inhibition, and thus could allow excess excitatory activity in the brainstem or in the gastrointestinal tract, which may promote the appearance of hyperemesis. Indeed, blockade of presynaptic cannabinoid CB_1_ receptors by rimonabant in Δ^9^-THC-tolerant dogs causes profound vomiting [26].

### 3.4. Acute vs. Chronic Cannabis Exposure

Published clinical and animal results from acute studies suggest that chronic exposure to Δ^9^-THC may not be necessary for the induction of emesis. In fact, acute intravenous injection of a crude marijuana extract in a single volunteer [44], or acute oral administration of dronabinol (Δ^9^-THC) in 3–30% of patients, have been shown to cause severe nausea, vomiting, diarrhea or crampy abdominal pain [45,46,47,48]. If the latter acute symptoms also represent components of cannabis hyperemesis syndrome, then chronic exposure to cannabinoids is not a necessary prerequisite for the induction of vomiting but may be needed for the intensification and cyclic nature of hyperemesis. Published results from animal models of emesis are in support of the latter proposal since acute intravenous or intraperitoneal administration of Δ^9^-THC can produce vomiting in naive dogs [49] or in 20–30% of naïve least shrews [35], while severe emesis is observed when Δ^9^-THC-dependent chronically-exposed dogs were given a small dose of the CB_1_ antagonist, rimonabant [26]. Overall, the discussed basic and clinical findings suggest that in a number of emetic species including humans, acute Δ^9^-THC administration in some susceptible individuals may induce a mild form of emesis, while chronic Δ^9^-THC exposure can cause severe hyperemetic syndrome. The reason(s) for Δ^9^-THC causing vomiting in a few drug naïve and apparently normal individuals, but not in all patients or test animals, still remains to be fully answered. One possibility is that some experimental subjects are particularly sensitive to certain actions of Δ^9^-THC such as mobilization of release of endocannabinoids and/or inflammatory mediators, such as arachidonic acid or their downstream metabolites with proemetic effects. Indeed, Δ^9^-THC has been shown to stimulate mobilization of arachidonic acid and anandamide release in different peripheral and central cell lines [50,51]. In fact both arachdonic acid and one of its major precursors, the endocannabinoid 2-AG, are potent emetogens when administered exogenously [52]. In addition, some of their downstream metabolites, such as prostaglandins [53] and cysteinyl leukotrienes [54] behave as potent emetogens. The enzyme responsible for the conversion of arachidonic acid to prostaglandins is cyclooxygenase, inhibition of which by indomethacin prevents the emetic ability of both arachidonic acid and 2-AG [52]. Moreover, 2-AG also mobilizes arachidonic acid release through activation of the phospholipase A_2_-prostanoid cascade via stimulation of cannabinoid CB_1_ receptors [55]. Thus, depending upon the degree of mobilization of such emetic mediators by Δ^9^-THC, one could account for the occurrence of mild emesis during the acute cannabis intake, while hyperemesis would be expected following chronic exposure to high doses of the euphoriant. A second possibility for Δ^9^-THC induction of vomiting in some but not all drug-naïve individuals would be the aforementioned genetic variation in accumulation of emetic metabolites or other emetic components present in cannabis. 

### 3.5. Constitutive CB_1_ Receptor Activity and Endogenous Inverse Agonists

Biochemical and behavioral studies have revealed that the efficacy of structurally diverse ligands for cannabinoid CB_1/2_ receptors vary and individual compounds may behave as partial agonists, full agonists, silent antagonists, partial inverse agonists or full inverse agonists, in a manner similar to the well known spectrum of benzodiazepine 1 receptor ligands for the GABA_A_-benzodiazepine chloride-ion-channel complex. Indeed, in the latter system, benzodiazepines such as diazepam act as full agonists by binding to the modulatory site of the ion-channel complex and enhance the inhibitory actions of GABA, those that decrease the actions of GABA via the same site are termed inverse agonists (e.g., diazepam binding inhibitor protein produced within the brain), while those compounds that have no effect on GABA inhibition are termed silent antagonists (e.g., flumazenil) [56]. Thus, diazepam-like full agonists produce anxiolytic and anticonvulsant effects, while inverse agonists induce opposite actions such as anxiety and convulsion. Flumazenil-like compounds are relatively silent antagonists and lack major direct effects but can antagonize the effects of both agonists and inverse agonists. It is important to recognize that according to the two-state receptor model of agonist action [27], receptors in a given tissue can exist in equilibrium between an active and an inactive conformational state, and in some tissues a small portion of the total receptor population exists in the active state and elicits cellular responses in the absence of its agonist. These are therefore recognized as constitutively active receptors [57]. Thus, constitutively active receptors turn on their signal transduction (e.g., activation of adenylate cyclase, opening or closing of an ion channel) and produce a small but measurable amount of basal activity in the absence of corresponding agonists. In such a system, full agonists act by preferentially binding to and maximally enriching the active receptor conformation, thereby maximally increasing effector activity (signal transduction). Partial agonists show a weaker preference for the active receptor conformation and shift the equilibrium to a smaller extent, and relative to full agonists produce submaximal effects, even when receptors are fully occupied. Inverse agonists bind preferentially to the inactive form of the conformational state of the receptor, leading to a reduction in basal effector activity. Silent antagonists bind equally well to both receptor conformations and thus do not alter the equilibrium between the two states and therefore do not alter signal transduction activity. However, such neutral antagonists can block the action of both agonists and inverse agonists. The biochemical and/or behavioral inverse agonist activity of a ligand can only be observed when there is a significant level of constitutive activity within the test system. In the cannabinoid system, although rimonabant can be classified as a CB_1_ receptor antagonist, significant published biochemical and behavioral literature suggest that it is not a silent antagonist but has a significant inverse agonist activity [43]. Interestingly and more recently a peptide (hemopressin) produced within the body has been identified as the first potent endogenous antagonist/inverse agonist of cannabinoid CB_1_ receptors [58]. In terms of nausea and emesis, rimonabant has been found to induce intense vomiting in drug naïve least shrews [18] and ferrets [43] as well as causing condition gaping in rats, an indicator of nausea and food-related malaise [59,60,61]. Furthermore, human subjects receiving rimonabant in clinical trials have reported adverse gastrointestinal events (such as nausea, vomiting and diarrhea), and such individuals have discontinued treatment more often than those given placebo [62,63]. Overall, the discussed findings suggest agonist activity of cannabinoid CB_1_ receptor ligands impart antiemetic activity and thus an endogenous cannabinoid antiemetic tone may exist, while the activity of the endogenous inverse agonist could lead to emesis. Supporting evidence comes from preliminary findings that, unlike rimonabant, the silent cannabinoid CB_1_ receptor antagonist with no inverse-agonist activity (AM4113) lacks emetic efficacy in the ferret [43]. Furthermore, a combination of anandamide and URB597 (an inhibitor of the metabolic enzyme (FAAH) for endocannabinoids) can attenuate emesis caused by cisplatin in the house musk shrew [60], while the selective reuptake inhibitor of the endocannabinoid system, VDM11 can attenuate apomorphine-induced emesis in least shrews [64]. However, as discussed earlier, when exogenously administered endocannabinoids are administered intraperitoneally by themselves (e.g., low doses of 2-AG or a 10 mg/kg dose of anandamide), they can be rapidly metabolized to their downstream emetic products and produce vomiting via other mechanisms [52]. The picture becomes even more complicated since VDM11 or URB597 failed to prevent vomiting caused by cisplatin in the least shrew [64], while cisplatin has been shown to increase brain tissue levels of 2-AG and concomitantly attenuate intestinal tissue levels of both 2-AG and anandamide in this species [64].

### 3.6. Brainstem vs. Enteric Emetic Loci

Since functional pathophysiology of nausea and emesis indicate these processes are controlled by a balance between the enteric and central nervous system [17], the enteric effects of cannabis (e.g., decreased gastrointestinal motility) are thought to override its brainstem-mediated antiemetic effects, to promote emesis [1,10,13]. However, this hypothesis does not reflect the current depth of knowledge on the antiemetic mechanisms of cannabinoids. In fact, through presynaptic CB_1_ receptor activation, Δ^9^-THC can inhibit intestinal contractile activity directly by reducing excitatory myenteric neurotransmission to the smooth muscle [65], whereas inhibition of gastric motility by Δ^9^-THC is primarily due to activation of CB_1_ receptor in the vagal circuitry of the brainstem [66]. Indeed, cannabinoids modulate emesis via activation of presynaptic CB_1_ receptors not only by modulating vagal afferent activity at three possible sites within the dorsal vagal complex nuclei of the brainstem {(i) vagal afferent terminals present in the nucleus of solitary tract (NTS) and dorsal motor nucleus of the vagus (DMNX); (ii) terminals of inhibitory interneurons in the NTS, and (iii) terminals of NTS neurons that project to the DMNX and area postrema (AP)}, but also via vagal efferents, since gastric motor inhibition caused by Δ^9^-THC can be abolished by vagotomy [17]. Vagal afferents have their cell bodies in the DMNX and project to both submucosal and myenteric plexi and their terminals contain CB_1_ receptors [67]. The main neurotransmitter in these nerves is acetylcholine, which influences motility, secretion and blood flow by interacting with enteric nerves. Thus, cannabinoid agonists are potent inhibitors of gastrointestinal tract motility, and inhibition of motility from stomach to colon occurs primarily via activation of presynaptic CB_1_ receptors under physiological conditions. This reduction in peristalsis may contribute to the peripheral component of antiemetic action of cannabinoids [68]. Moreover, cannabinoids inhibit the relaxation of lower esophageal sphincter (normally an effect necessary for emesis to occur) via the brainstem, which further accounts for Δ^9^-THC’s antiemetic actions [66,69]. Although cannabinoid CB_2_ receptors do not appear to affect gut motility under physiological circumstances, they potentially regulate motility in pathophysiological states, which could also account for the antiemetic activity of cannabis [17].

### 3.7. Hot Bathing

The compulsive “hot bath (shower) behavior” has also been implicated in cannabinoid hyperemesis syndrome and has been suggested to occur via “disequilibrium of the thermoregulatory system of the hypothalamus” which could settle with hot baths or showers [1]. Furthermore, Chang and Windish [8] have suggested that the desire for hot showers is either to counteract the cannabis-induced decrease in core body temperature or is a direct response to CB_1_ receptor activation in the hypothalamus. In fact the medial preoptic/anterior hypothalamic area (POA) is the primary thermosensitive site of the CNS [70] and is particularly enriched with cannabinoid CB_1_ receptors [71]. Furthermore, it is well known that in animals peripheral administration of large doses of phyto-, synthetic or endocannabinoids cause hypothermia via a reduction in core body temperature through stimulation of cannabinoid CB_1_ receptors [72,73]. Another possible mechanism via which large doses of Δ^9^-THC could induce hypothermia is via the discussed Δ^9^-THC-induced liberation of endocannabinoids and/or their downstream metabolites. Indeed, both anandamide and its major metabolite arachidonic acid reduce core body temperature which can be antagonized by the cyclo-oxygenase inhibitor, ibuprofen [72]. Irrespective of molecular mechanisms of the hypothermic response, a simple explanation of the self-learned “compulsive hot-bath” activity would be amelioration of the hypothermia induced by large doses of cannabis in a subset of metabolically compromised patients who chronically use this euphoriant. 

Despite the literature prominence of cannabinoid-induced hypothermia, the mechanisms underlying the effect and its relevance for human conditions remain to be established. Indeed, at lower human-relevant doses (<2 mg/kg), Δ^9^-THC raises the temperature in rodent’s core body, muscle and brain, while concomitantly decreasing tail skin temperature [73,74]. Furthermore, increases in brain and muscle temperatures are associated with an initial skin hypothermia reflecting peripheral vasoconstriction, which can be followed later by a rebound-like skin hyperthermia [75]. Therefore, a more logical association for the latter findings and the “compulsive bath activity” would be that, at human-relevant doses, cannabis would tend to raise the core body temperature while concomitantly reducing skin temperature, and the warm shower would help to increase blood flow to the skin to dissipate the raised core body heat. This hypothesis fits well with the thermoregulation physiology, as the PAO in the hypothalamus contains warm-sensitive, cold-sensitive, and temperature-insensitive neurons. The warm-sensitive neurons act as the central integrators of thermal information, as their activity is determined both by their own temperature and by afferents arising from skin and visceral thermoreceptors. Thus, if the warm-sensitive neurons are activated by cannabis-induced increase in core body temperature, they subsequently could trigger sympathetically-mediated responses to promote heat loss such as vasodilation and sweating [70]. Although most of the discussed cannabinoid hyperemesis reports do not indicate a significant change in body temperature, Allen and colleagues [1] did observe sweating in some individuals while two of the seven patients experienced pyrexia. The latter symptoms could be explained by the above-discussed rebound hyperthermia phenomenon. Lack of reported temperature changes in hyperemesis patients is not surprising since: (i) the core body temperature does not always correlate with temperatures in other body areas [76]; (ii) the skin temperature of patients may become normal after taking showers; (iii) reflective measurement of skin temperature is nearly impossible since it would depend on both the stage of hyperemesis and when the actual measurement was made.

## 4. Summary

The clinical occurrence of cannabis-induced hyperemesis in some patients who chronically use cannabis has been well established. Traditionally, the most psychoactive component of marijuana plant, Δ^9^-THC, and related cannabinoid CB_1_ receptor agonists are viewed as agonist antiemetics and are employed in the clinic for the prevention of chemotherapy-induced nausea and vomiting in cancer patients. However, published clinical literature indicates that in some prone individuals not only acute cannabis exposure can induce vomiting, but upon chronic cannabis intake the intensity of emesis strengthens and takes a cyclic nature in such individuals. These clinical findings are supported by published preliminary data in dogs and least shrew models of emesis. This review critically examines possible pharmacokinetic and pharmacodynamic mechanisms via which the enigmatic syndrome can occur.
